# Regional Variation in the Density of Essential Genes in Mice

**DOI:** 10.1371/journal.pgen.0030072

**Published:** 2007-05-04

**Authors:** Kathryn E Hentges, David D Pollock, Bin Liu, Monica J Justice

**Affiliations:** 1 Faculty of Life Sciences, The University of Manchester, Manchester, United Kingdom; 2 Department of Biochemistry and Molecular Genetics, University of Colorado Health Sciences Center, Aurora, Colorado, United States of America; 3 Department of Molecular and Human Genetics, Baylor College of Medicine, Houston, Texas, United States of America; Stanford University School of Medicine, United States of America

## Abstract

In most species, and particularly in vertebrates, the percentage of genes absolutely required for survival, the essential genes, has not been estimated. To obtain this estimation, we used the mouse as an experimental model to carry out high-efficiency N-ethyl-N-nitrosourea (ENU) mutagenesis screens in two balancer chromosome regions, and compared our results to a third previously published screen. The number of essential genes in each region was predicted based on allele frequencies. We determined that the density of essential genes differs by up to an order of magnitude among genomic regions. This indicates that extrapolating from regional estimates to genome-wide estimates of essential genes has a huge variance. A particularly high density of essential genes on mouse Chromosome 11 coincides with a high degree of regional linkage conservation, providing a possible causal explanation for the density variation. This is the first demonstration of regional variation in essential gene density in the mouse genome.

## Author Summary

The genome sequences of many organisms are now complete. However, speculation remains regarding the function of many newly discovered genes. There is also debate about the percentage of genes that are required to build an organism. These genes, which are necessary for the development of the organism, are essential genes. We have performed mutagenesis screens that allow the identification of mutations in essential genes from specific regions of the mouse genome. From these data we have predicted the number of essential genes in three regions of the mouse genome. When we compared these predictions, we found that the density of essential genes varies in different regions of the mouse genome. We then analyzed these regions of the genome to identify similar regions in other mammals. We found that regions of the mouse genome with a high density of essential genes are more similar to other species than those regions with fewer essential genes, suggesting that throughout evolution genomic regions with many essential genes remain intact.

## Introduction

In the era of complete genomes, the total number of genes in a sequenced organism can now be predicted, but the function and selective importance of a substantial fraction of genes remains unknown. Some gene functions may be of central importance to the organism, whereas other gene functions may be useful, but not critical, or may have functions that are partially redundant. Genes are classified as essential if an organism cannot develop to maturity without them. Here, employing balancer chromosome mutagenesis studies on specific regions of the mouse genome, we evaluate the distribution of essential genes in these regions. Our data also show that in mammals, similar to worms [1], essential gene clusters are located in genomic regions with high linkage conservation.

## Results

Essential genes in two genomic regions were targeted using balancer chromosome screens: a 35-Mb region of mouse Chromosome 11 between the *Trp53* and *Wnt3* loci [2] and a 20-Mb region of mouse Chromosome 4 between markers *D4Mit281* and *D4Mit51* [3]. For comparison, we also analyzed results from an earlier mutagenesis study that identified nine essential loci in a 20-Mb deletion region on mouse Chromosome 7 [4]. In our study, we considered essential genes to be those that when mutated cause lethality at or before birth. To improve the accuracy of the analysis, we performed pair-wise complementation tests of fully penetrant mutant lines from each screen to identify alleles at each locus. From 785 pedigrees bred in the Chromosome 11 balancer screen, we isolated 45 mutant lines that die at or before birth (Table 1). These 45 lines formed 40 complementation groups, and thus only five loci were detected more than once (Table 1). From 551 pedigrees bred in the Chromosome 4 balancer screen, we isolated 16 mutant lines that die at or before birth (Table 1). These mutants formed 12 complementation groups (Table 1). In comparison, the deletion screen on Chromosome 7 bred 4,557 pedigrees to generate 24 fully penetrant lethal mutant lines that fell into nine complementation groups [4]. Notably, only a third of the number of pedigree groups were screened on Chromosome 11 as compared to Chromosome 7. However, we obtained about two and a half times as many mouse lines carrying essential genes, and almost six times as many complementation groups.

**Table 1:**
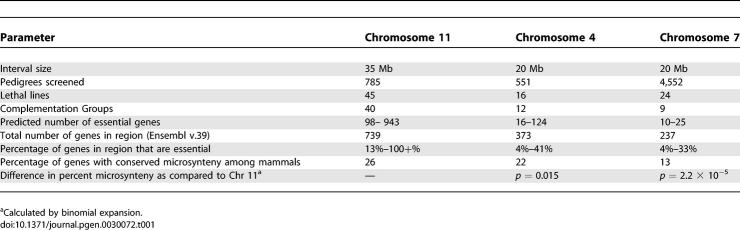
Essential Genes in Three Regions of the Mouse Genome

To predict the number of essential genes in each chromosomal region, we employed a Bayesian approach that incorporates variation in the degree of mutability among loci to provide a credible range of values rather than a point estimate [5]. This analysis requires knowledge of the number of complementation groups in each region, and cannot be applied to studies that fail to consider allelism. Evidential support for gamma and mixture models that incorporate variation in mutability among loci was minimal based on the datasets alone, although previous analyses show that variation in mutability is the norm [5]. When mutabilities vary, genes with low mutabilities tended to be under-counted if a model with a single mutability rate (Poisson) is assumed; the numbers of lethal mutations predicted from a Poisson distribution are therefore probably an underestimate [6,7]. To obtain an accurate measurement, we considered gamma-distributed mutabilities with the shape parameter constrained to reasonable values (*a* = 0.2–5.0) based on previous observations [5].

There were 222 essential genes (between 98 and 943 based on a very conservative 99% credible region) predicted in the Chromosome 11 balancer region (Figure S1A; Table S1). Similarly, 31 essential genes (16 to 124) were predicted in the Chromosome 4 balancer region (Figure S1B). The Chromosome 7 mutagenesis experiment was more highly saturated, with 12 essential genes estimated (10 to 25, Figure S1C).

These three regions clearly vary considerably in their density as well as their number of essential genes. The predicted mean density of essential genes per Mb in the Chromosome 11 balancer region is four times greater than the density on Chromosome 4, and 11 times greater than the density on Chromosome 7. All density differences between chromosomes are significant, and the chromosome 11/4 density ratio is at least 2.26 (*p* < 0.05), while the 11/7 ratio is at least 7.0 (*p* < 0.05). The number of essential genes predicted in each region is also significantly different (*p* < 0.05) as a proportion of the total number of predicted genes (739, 373, and 237, respectively).

The Chromosome 11 balancer region has unusually high synteny in addition to its high essential gene density: human Chromosome 17 is entirely conserved with this region of mouse Chromosome 11, making it the most conserved mouse–human autosomal linkage group (Figure S2). Chromosomes 4 and 7 have less synteny conservation with human chromosomes (data not shown). Although gene density (as well as essential gene density) is high on Chromosome 11, we found that on other mouse chromosomes the relationship between gene density and synteny conservation was weak (Figure S3).

The number of essential genes appears to be predictive of microsynteny and sequence conservation as well as large-scale synteny. We examined homologs among mouse, rat, human, dog, and cow to determine which genes had the same neighbors in all five species, and found that 26% of the genes on mouse Chromosome 11 had conserved microsynteny. In contrast, only 22% of the genes on Chromosome 4 and 13% of the genes on Chromosome 7 had conserved microsynteny in all five species (Table 1). These frequency differences are significant (Table 1). At the sequence level, a previous comparison between the C57BL/6J and 129S5 mouse strains demonstrated that Chromosome 11 has much higher sequence conservation than Chromosomes 4 or 7 [8]. Overall, Chromosome 11 is the third most-conserved chromosome between these two strains [8].

## Discussion

In this first comparative study of essential gene densities in a mammalian genome, we have identified surprising differences as large as an order of magnitude. Our region-specific mutagenesis screens combined with complementation testing were laborious but necessary for these calculations. Our statistical accommodation of variation in mutability, although more complex than most previous studies, allowed a more accurate assessment of the variability in essential gene density.

Sequence conservation of regions dense in essential genes is perhaps not surprising, but synteny conservation is more so. A weak correlation between essential gene density estimates and synteny was previously observed in roundworms based on RNAi [1], but our observations in mammals use a more precise assessment of essential function and a more definitive assessment of large-scale synteny among more species, as well as an assessment of microsynteny. Thus, it is reasonable to consider a general causal relationship between essential genes and reduced rates of chromosomal translocation and rearrangement. If adjacent essential genes generally reduce the probability of productive chromosomal translocations between them, essential gene-dense regions would be expected to expand over time as essential genes randomly join a cluster, but then have a reduced probability of departing. Thus, it appears that the large number of densely packed essential genes on the balancer region of mouse Chromosome 11 may have forced it to remain as a unit in spite of millions of years of divergence and speciation. This also predicts that syntenically conserved regions should be especially attractive targets for future essential gene detection.

It is traditional to use regional estimates of essential gene density to estimate the total number of essential genes in the genome. If we extrapolate the number of essential genes as a proportion of predicted genes in each region, there would be 5,749 essential genes overall (20% of the genome). If we extrapolate based on the density of essential genes per Mb, we predict about twice as many (10,849). The results of our own research, however, indicate that the variability on this extrapolation is huge. If the variability of the regional estimates, as well as the variability among the regional estimates (up to 11-fold), is taken into account, the estimate ranges from ∼1,100 essential genes up to more genes than the total predicted number of genes in the genome (28,594). It is a near certainty that such variability is not specific to our study, but applies to all previous estimates of essential genes that utilized one or a few genomic regions. If the relationship between essential genes and synteny, particularly microsynteny, is consistently upheld in a variety of organisms, more accurate and believable estimates could be obtained by using microsynteny and conservation in essential gene predictions.

## Materials and Methods

### Saturation calculation.

The fraction of lethal mutations remaining to be isolated from each screen was calculated using Saturate [5]. We considered gamma-distributed mutabilities with the shape parameter constrained to reasonable values (*a* = 0.2–5.0) based on previous observations. For the gamma model, alpha was constrained to be less than 5.0.

### Sequence comparisons.

Genomic sequences of mouse, human, chimp, rat, cow and dog were downloaded from Ensembl v.38 (http://www.ensembl.org/info/data/download.html). Each region of mouse sequences was divided into 150-kb fragments, which were then blasted using Megablast (http://www.ncbi.nlm.nih.gov/BLAST/download.shtml). The sequence comparison was carried out on a Sun cluster with SunFire 280R (http://www.sun.com). Mouse genomic annotation was downloaded from Ensembl BioMart v.38 (http://www.ensembl.org/Multi/martview). To visualize the blast results, we developed in-house software written in Microsoft Visual Basic (http://www.microsoft.com). All blast results were uploaded in a MS SQL server database, and the results displayed on a PC. Microsynteny comparisons were performed using gene annotation from Ensembl Biomart v.38. A list of genes with conserved microsynteny will be provided upon request.

### Essential gene calculations.

An explanation of calculations is found in Table S2. All predictions are based on protein-coding known genes found in Ensembl Biomart v.39. The extremes of two distributions such that they were as similar as possible but the joint probability was no less than 5% was taken to obtain the minimal ratio of the two essential gene predictions. In no case did the density distributions overlap with greater than 5% probability.

## Supporting Information

Figure S1 Prediction of Essential Genes These results allowed for variable mutation rates among genes. The fraction of essential genes not yet discovered in each screen is shown. The *y-*axis gives probability (percent), and the *x-*axis shows the fraction of lethal complementation groups undiscovered.
(A) The Chromosome 11 balancer screen. The 99% credible region predicts that 59%–96% of the essential genes have not been isolated in the screen.
(B) The Chromosome 4 balancer screen. The 99% credible region predicts that 27%–90% of the essential genes have not been isolated in the screen.
(C) The Chromosome 7 deletion screen. The 99% credible region predicts that 6%–64% of the essential genes have not been isolated in the screen.
(1.7 MB JPG)

Figure S2 Genomic Comparisons of mouse Chromosome 11 Balancer Region Mouse genomic sequences from the balancer region were compared with genome sequence from human, chimp, rat, dog, and cow.
(A) Conservation between the mouse Chromosome 11 balancer interval and human Chromosome 17.
(B) Conservation between the mouse Chromosome 11 balancer interval and chimp Chromosome 19.
(C) Conservation between the mouse Chromosome 11 balancer interval and rat Chromosome 10.
(D) Conservation between the mouse Chromosome 11 balancer interval and dog Chromosomes 9 and 5. Note that the break in synteny is in a gene-poor region of mouse Chromosome 11.
(E) Conservation between mouse Chromosome 11 and cow Chromosomes 19 and 23. Again, a break in synteny occurs in a gene-poor region. Note the many inversions between mouse Chromosome 11 and cow Chromosome 19, which is currently available only as shotgun sequence.
(13.8 MB TIF)

Figure S3 Comparison of Conservation between Mouse and Human for a Gene-Dense and Gene-Poor Region (A) Conservation between proximal mouse Chromosome 17 (11–46 Mb) and human Chromosomes 6 and 21. This region of mouse Chromosome 17 is predicted to contain 636 protein-coding known genes in 35 Mb. There is less linkage conservation than in the Chromosome 11 balancer region.
(B) Conservation between distal mouse Chromosome 12 (71–92 Mb) and human Chromosome 13. This region of mouse Chromosome 12 is predicted to contain 157 protein-coding known genes in 21 Mb (Ensembl v.38), and shows high linkage conservation.
(3.4 MB TIF)

Table S1 Parameter Estimates for Saturation Analysis Predictions for the percent essential genes remaining undiscovered, along with other model parameters, are shown for the Poisson (single mutation rate) and gamma (variable mutation rate) analyses. Only the rate (for the Poisson) and the shape parameters (alpha and beta, respectively, for the gamma analysis) are free parameters, while the undiscovered loci estimates and the rate estimate (for the gamma analysis) are calculated from other parameters. Credible regions shown are 95% and 99% for the Poisson model and 99% for the gamma model. For the gamma model, alpha was constrained to be less than 5.0. The maximum log likelihood scores for each analysis are also shown.
(37 KB XLS)

Table S2 Calculations of Essential Genes The lower limit and upper limit of essential genes in each region predicted from each statistical model is shown in the chart. An explanation of the calculations for essential genes for each chromosome interval and for the whole genome is also provided.
(120 KB DOC)
