# Supplementary material for: Regional Variation in the Density of Essential Genes in Mice
Source: PLoS Genet. 2007 May 4;3(5):e72. doi: 10.1371/journal.pgen.0030072 (PMC1865562; doi:10.1371/journal.pgen.0030072)
Supplement: Supplementary file 1 [file pgen.0030072.st002.doc]

**Supporting Table 2.** **Calculations of essential genes**

The lower limit and upper limit of essential genes in each region predicted from each statistical model is shown in the chart. An explanation of the calculations for essential genes for each chromosome interval and for the whole genome is also provided.

| Chromosome  Interval | Prediction | Total genes (Ensembl 39) | Complementation groups identified | Lower limit essential genes | Upper limit essential genes | Maximum percent essential genes |
| --- | --- | --- | --- | --- | --- | --- |
| Chromosome 7 Deletion | Poisson | 237 | 9 | 9 | 12 | 5.0 |
| Chromosome 7 Deletion | Gamma | 237 | 9 | 10 | 25 | 10.4 |
| Chromosome 4 Balancer | Poisson | 373 | 12 | 16 | 55 | 14.9 |
| Chromosome 4 Balancer | Gamma | 373 | 12 | 16 | 124 | 33.2 |
| Chromosome 11 Balancer | Poisson | 739 | 40 | 97 | 399 | 54.0 |
| Chromosome 11 Balancer | Gamma | 739 | 40 | 98 | 943 | 127.6 |

Essential gene calculations:

Chromosome 11:

Gamma prediction shows 59.28 – 95.76% of the essential genes have not been isolated in the screen (Figure 1, Supplemental Table 1). 40 complementation groups have been isolated. If 59.28 – 95.76% are not yet identified, then these 40 groups represent 4.24 – 40.72% of the essential genes in the region. If 40 groups represent 40.72% of the groups in the region, then the total number of groups is 98 (40/0.4072 = 98). If 40 groups represent 4.24% of the groups in the region, then the total number of groups is 943 (40/0.0424 = 943). The lower percentage of essential genes for this region is 98/739 = 13.2%. Note that Ensembl predicts only 739 genes in region, so we have considered the upper limit of essential genes as 100% of genes. If mutations in the same gene complement, then the number of essential genes as predicted from the number of lethal complementation groups can exceed the number of genes annotated in the region, although we have no experimental data to confirm that any separate complementation groups are different mutations in the same gene. There is experimental evidence to suggest that there are dominant lethal mutations on chromosome 11, as deletions of sub-regions within the balancer interval are lethal in the hemizygous state (Hentges et al., 2006). Dominant lethal mutations would not be recovered from our mutagenesis screen, and could not be complementation tested. Calculations for the number of essential genes in each other genomic interval were calculated in the same manner based on gamma predictions for each genomic interval.

Total genome calculations:

Estimates of the total number of essential genes in the genome were calculated based on their proportion of the genes in each region, or per Mb. Minima and maxima were taken based on the weighted average of the minima and maxima for the gene regions combined, while extreme minima and maxima considering the variability among regions were taken as the minima and maxima across the three regions if each region were assumed to be an exemplar of the whole genome (as is commonly done in most studies of individual regions).
